# Excessive Gestational Weight Gain Alters DNA Methylation and Influences Foetal and Neonatal Body Composition

**DOI:** 10.3390/epigenomes7030018

**Published:** 2023-08-16

**Authors:** Perla Pizzi Argentato, João Victor da Silva Guerra, Liania Alves Luzia, Ester Silveira Ramos, Mariana Maschietto, Patrícia Helen de Carvalho Rondó

**Affiliations:** 1 Nutrition Department, School of Public Health, University of São Paulo, Avenida Dr. Arnaldo 715, São Paulo 01246-904, SP, Brazil; perlapizzi@usp.br (P.P.A.); lianialuzia@usp.br (L.A.L.); 2 Brazilian Biosciences National Laboratory (LNBio), Brazilian Centre for Research in Energy and Materials (CNPEM) and Graduate Program in Pharmaceutical Sciences, Faculty of Pharmaceutical Sciences, University of Campinas, Rua Giuseppe Máximo Scolfaro 10.000, Cidade Universitária, Campinas 13083-970, SP, Brazil; jvsguerra@gmail.com; 3 Department of Genetics, Ribeirão Preto Medical School, University of São Paulo, Avenida Bandeirantes 3900, Ribeirão Preto 14049-900, SP, Brazil; eramos.epigen@gmail.com; 4 Boldrini Children’s Hospital, University of Campinas, Rua Márcia Mendes 619, Cidade Universitária, Campinas 13083-884, SP, Brazil; marianamasc@gmail.com

**Keywords:** gestational weight gain, DNA methylation, ultrasonography, plethysmography, offspring body composition

## Abstract

Background: Changes in body weight are associated with the regulation of DNA methylation (DNAm). In this study, we investigated the associations between maternal gestational weight gain-related DNAm and foetal and neonatal body composition. Methods: Brazilian pregnant women from the Araraquara Cohort Study were followed up during pregnancy, delivery, and after hospital discharge. Women with normal pre-pregnancy BMI were allocated into two groups: adequate gestational weight gain (AGWG, *n* = 45) and excessive gestational weight gain (EGWG, *n* = 30). Foetal and neonatal body composition was evaluated via ultrasound and plethysmography, respectively. DNAm was assessed in maternal blood using Illumina Infinium MethylationEPIC BeadChip arrays. Linear regression models were used to explore the associations between DNAm and foetal and neonatal body composition. Results: Maternal weight, GWG, neonatal weight, and fat mass were higher in the EGWG group. Analysis of DNAm identified 46 differentially methylated positions and 11 differentially methylated regions (DMRs) between the EGWG and AGWG groups. Nine human phenotypes were enriched for these 11 DMRs located in 13 genes (*EMILIN1*, *HOXA5*, *CPT1B*, *CLDN9*, *ZFP57*, *BRCA1*, *POU5F1*, *ANKRD33*, *HLA-B*, *RANBP17*, *ZMYND11*, *DIP2C*, *TMEM232*), highlighting the terms insulin resistance, and hyperglycaemia. Maternal DNAm was associated with foetal total thigh and arm tissues and subcutaneous thigh and arm fat, as well as with neonatal fat mass percentage and fat mass. Conclusion: The methylation pattern in the EGWG group indicated a risk for developing chronic diseases and involvement of maternal DNAm in foetal lean and fat mass and in neonatal fat mass.

## 1. Introduction

Weight gain during pregnancy is important for adequate development of the foetoplacental unit. The Institute of Medicine (IOM) recommends gestational weight gain (GWG) [1] based on pre-gestational body mass index (BMI). Excessive GWG is associated with tiredness, altered breathing, joint alterations, maternal obesity, caesarean section, obstetric risks, and postpartum weight retention [2].

Overweight is a global problem in women of childbearing age. In the United States, maternal obesity and excessive GWG affect approximately 60% of women [3]. About 30% of women in Europe and 10% of women in Asia who become pregnant are overweight or obese [4]. In Brazil, a study using weight data of 840,243 women from the Food and Nutritional Surveillance System showed an increase in overweight and pre-gestational obesity, as well as in the prevalence of excessive GWG, in 11 of the 27 units of the Brazilian federation between 2008 and 2016 [5].

Overweight or obesity during pregnancy contributes to the development of diseases in the offspring at different stages of life. This fact was explained by foetal metabolic programming, a process describing the epigenetic mechanisms that modulate gene expression [6,7]. One such mechanism is DNA methylation (DNAm). During pregnancy, maternal diet, smoking, stress and hormonal changes affect DNAm patterns [8]. Maternal obesity, which is associated with birth weight, also alters the methylation of CpG sites (CpGs) [9]. However, little is known about the effect of excessive GWG in the absence of maternal obesity on DNAm and neonatal body composition. Therefore, the objective of the present study was to assess changes in maternal DNAm related to GWG in women who started pregnancy with an adequate BMI and their associations with foetal and neonatal body composition.

## 2. Materials and Methods

### 2.1. Subjects

This is a prospective cohort study involving healthy pregnant women randomly selected from 34 Basic Health Units and the Municipal Maternity Hospital of Araraquara, São Paulo, Brazil, as part of the epidemiological Araraquara Cohort Study. A convenience sample of pregnant women with a normal pre-pregnancy BMI (≥18.5 and <24.9 kg/m^2^) was randomly selected and further divided into two groups according to GWG recommended by the IOM [1]: excessive gestational weight gain (EGWG; total weight gain > 16 kg; *n* = 30) and adequate gestational weight gain (AGWG; 11.5 kg > total weight gain < 16.0 kg; *n* = 45).

The women were followed up at three different time points during pregnancy, at delivery, and after hospital discharge: T1, up to gestational age (GA) ≤ 15 weeks; T2, 20–26 weeks; T3, 30–36 weeks; T4, at delivery, and T5, hospital discharge (72 h after delivery). This study was conducted in accordance with the Declaration of Helsinki and approved by the Research Ethics Committees of the School of Public Health, University of São Paulo (protocol number 2.570.576).

Pregnant women who met one of the following exclusion criteria were removed from further analyses: more than 15 gestational weeks; under 18 and over 35 years of age; diagnosis of chronic diseases, severe mental illness, and infectious disease; multiple pregnancy; a history of abortion; smoking and use of alcohol or other drugs at the beginning of the study or during follow-up. Women who lost weight or had poor weight gain during pregnancy, those who had a stillborn child or a child with congenital diseases, and those who failed to attend one appointment during the follow-up period were also excluded.

### 2.2. Anthropometric Assessment of the Pregnant Women

Pre-gestational maternal BMI was used for nutritional diagnosis, identifying pregnant women with normal BMI. The pre-gestational weight was measured until the 13th week of gestation (assessed via ultrasonography). Weight at the three different time points during pregnancy and at delivery was measured via bioimpedance analysis using the Inbody 370 analyser (Biospace^®^, Seoul, Republic of Korea). Women were classified according to the GWG recommendations of IOM [1] as EGWG and AGWG.

### 2.3. Foetal Body Composition

Foetal body composition was evaluated via ultrasonography at T2 and T3. A trained sonographer performed the measurements using the ACUSON X300TM ultrasound system, premium edition (Siemens^®^, Mountain View, CA, USA) equipped with curvilinear abdominal transducers (C5-2, C6-3, V7-3). The following foetal parameters were assessed: subcutaneous abdominal fat thickness (SCFT, mm); total thigh tissue = total muscle mass + fat (cm^3^); thigh muscle mass = internal area of the subcutaneous tissue of the thigh (cm^3^); subcutaneous thigh fat = total thigh tissue − thigh muscle mass (cm^3^); total arm tissue = thigh muscle mass + fat (cm^3^); arm muscle mass = internal area of the subcutaneous tissue of the arm (cm^3^); and subcutaneous arm fat = total arm tissue − arm muscle mass (cm^3^).

### 2.4. Anthropometry and Body Composition of Neonates

Neonates were evaluated at hospital discharge (T5), 12–72 h after delivery. Length (cm) was measured with a Seca^®^ 416 infantometer (Seca^®^, Hamburg, Germany). The body composition and weight of the neonates were evaluated via air displacement plethysmography using the PEA POD equipment (Cosmed^®^, Concord, CA, USA).

### 2.5. Sample Collection and DNA Extraction

At T3, 2 mL of maternal blood during fasting was collected into VACUETTE^®^ EDTA tubes, manually homogenised, and refrigerated for further extraction of DNA. Total genomic DNA was extracted from maternal blood samples with proteinase K (Thermo Fisher^®^ Products, Vilnius, Lithuania) according to the manufacturer’s protocol, followed by a modified salting method [10]. The extracted DNA was quantified in a Nanodrop spectrophotometer (Thermo Fisher Scientific Inc., Santa Clara, CA, USA). Samples with an OD260:OD280 ratio greater than 1.8 and an OD260:OD230 ratio between 1.8 and 2.2 were considered to be pure. Integrity was checked using 2.0% agarose gel electrophoresis with ethidium bromide diluted to a concentration of ~50 ng/µL. The DNA methylome was evaluated in eight pregnant women of each group, and matched for baby’s sex and maternal parity.

### 2.6. Methylation Analysis

Sixteen pregnant women (AGWG, *n* = 8 versus EGWG, *n* = 8) were selected as a convenience sample for DNA conversion high-quality bisulfite-converted (EZ DNA Methylation Kit, Zymo Research Corp., Irvine, CA, USA) that were hybridized to the Infinium HumanMethylationEPIC BeadChip microarray (EPIC, Illumina, San Diego, CA, USA), following the Illumina Infinium HD protocol at Diagenode (www.diagenode.com, accessed on 24 May 2023). A total conversion rate with bisulfite of 95.60% was found. Raw data were extracted as IDAT files with the iScan SQ Scanner (Illumina) using the GenomeStudio software (v.2011.1) and the methylation module v.1.9.0 (Illumina). Probes were annotated according to the Illumina annotation file using the Human GRCh37/hg19 assembly.

Quality control was assessed on the IDAT files, and loaded into the R environment with the ChAMP package [11]. Failed probes (detP > 0.01, *n* = 3755), probes with <3 beads in at least 5% of samples (*n* = 40,386), non-CG probes (*n* = 2791), multi-hit probes [12] (*n* = 11), and probes located in XYS [13] (*n* = 109,529) were excluded. The remaining 709,466 probes were normalised using the BMIQ method [14] (Figure S1). Singular value decomposition (SVD) analysis [15] identified batch effects in the dataset, which were corrected [16]. Biological covariates were then correlated with the main components of the methylation data. Next, we estimated the influence of methylation resulting from the distinct cellular composition of whole blood using methylation profiles of the major blood cell types. Based on the results, we adjusted the cell-type heterogeneity for each sample using the RefbaseEWAS method [17]. Methylation levels for each probe are reported as beta-values (0: unmethylated, 1: methylated), which were used for graphical representation; M-values (logit-transformed beta-values) were used for statistical analysis due to the homoscedastic behaviour of the data, unless otherwise stated.

Differential methylation analyses were performed comparing the two groups of pregnant women, AGWG and EGWG. Empirical Bayesian estimation was applied to the M-values using a linear regression model from the limma package [18] to identify the differential methylated positions (DMPs). The bumphunter algorithm [19] was used to identify differentially methylated regions (DMRs), which are extended segments of the genome displaying quantitative alterations in DNA methylation levels between the two groups, AGWG and EGWG. The algorithm considered a minimum of 7 CpGs within a maximum gap of 300 bases, applied lowess smoothing of the genomic profile, and employed 250 resamples to compute the null distribution. To further analyse the DMRs, we calculated the mean beta-value for each DMR, based on the methylation levels of the CpG sites within it, enabling us to determine the methylation status of these regions. Subsequently, a heatmap was generated to visualise the identified DMRs and their methylation patterns. Functional annotation of DMRs was performed via enrichment analysis using GREAT [20]. We considered DMPs, DMRs, and functional annotation with a *p*-value ≤ 0.05 to be significant.

### 2.7. Data Analysis

The Shapiro–Wilk test was applied to test the normality of the data. The *t*-test for independent samples was used for comparisons between the AGWG and EGMG groups. The chi-square test was applied to compare categorical variables between the two groups of pregnant women. Repeated measures ANOVA using a mixed model and Bonferroni’s post hoc test were performed, in which the follow-up data were the repeated measures over time and the groups were the independent variables. Univariate and multiple linear regression models were used to explore the associations between mean maternal DNAm levels in each DMR (using the mean beta-value of the region) and markers of foetal and neonatal body composition. The outcome measures were weight, SCFT, total thigh tissue, thigh muscle mass, subcutaneous thigh fat, total arm tissue, arm muscle mass and subcutaneous arm fat of the foetus at T2 and T3, and weight, length, fat-free mass percentage, fat mass percentage, and fat-free mass and fat mass of the neonate at T5. The confounding variables included maternal age, pre-pregnancy BMI, GWG, GA, and newborn sex. Statistical significance was set at *p* ≤ 0.05 and analysis was performed using the SPSS 18.0 software (SPSS, Chicago, IL, USA).

## 3. Results

### 3.1. Characteristics of the Pregnant Women and Their Neonates

Table 1 shows the demographic, socioeconomic and obstetric characteristics of the pregnant women. No differences in age, ethnicity, marital status, or education were found between the groups.

### 3.2. Anthropometry and Body Composition of the Pregnant Women and of Their Foetuses and Neonates

**Figure 1:**
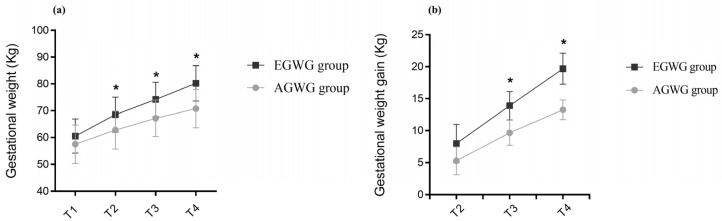
(**a**,**b**) Evolution of gestational weight and gestational weight gain. Repeated measures ANOVA using a mixed model and Bonferroni’s post hoc test. T1 = ≤15 gestational weeks, T2 = 20–26 weeks, T3 = 30–36 weeks and T4 = delivery. * *p* ≤ 0.05.

The anthropometric characteristics and body composition of pregnant women in the AGWG and EGWG groups are shown in Table 2. As expected, pre-gestational weight, pre-gestational BMI, height or pre-gestational body composition did not differ between AGWG and EGWG. However, total GWG was significantly higher in EGWG (*p* = 0.01). Furthermore, significant differences in weight and GWG between the two groups occurred with advancing gestation. There was an effect of time [F(3;219) = 1484.79; *p* < 0.001], with T4 > T3 > T2 > T1 (*p* < 0.001), and group [F(1;73) = 15.95; *p* < 0.001] on weight, with EGWG women exhibiting significantly higher weights than AGWG women (*p* < 0.001). There was also an effect of the time*group interaction [F(3;219) = 53.50; *p* < 0.001] on weight, where AGVW = EGWG, with T4 > T3 > T2 > T1 (*p* < 0.001). Comparison of the different time points showed no significant difference between the groups at T1 (*p* = 0.07), but there were statistically significant differences at T2, T3, and T4. With the exception of T1, the EGWG group always had significantly higher weights than the AGWG group (*p* < 0.001), Figure 1a. There was an effect of time [F(2;146) = 894.63; *p* < 0.001], with T4 > T3 > T2 (*p* < 0.001), and group [F(1;73) = 101.96; *p* < 0.001] on GWG, with significantly higher GWG in the EGWG group (*p* < 0.001). There was also an effect of the time*group interaction [F(2;146) = 32.24; *p* < 0.001] on GWG, where AGWG = EGWG, with T4 > T3 > T2 (*p* < 0.001). Comparison of the different time points showed statistically significant differences at T2, T3, and T4. Gestational weight gain was always higher in the EGWG group compared to the AGWG group (*p* < 0.001), Figure 1b.

Table 2 also shows the foetal body composition at T2 and T3 and anthropometry and neonatal body composition at T5. There were no differences in the foetal body composition parameters investigated. However, neonates of the EGWG group at 72 h of life had higher weights (*p* = 0.027) and fat mass (*p* = 0.039) than those born to AGWG mothers.

### 3.3. Characterization of DNA Methylation

DNA methylation at 709,466 CpGs was evaluated in the two groups of pregnant women. Multidimensional scaling (MDS) analysis of the 1% most variable positions showed inter-sample variability, indicating that there were no systemic methylation changes among groups (Figure S2). To obtain more robust findings and considering the small sample size in our study, we searched for DNAm differences using two approaches: DMPs and DMRs.

**Figure 2:**
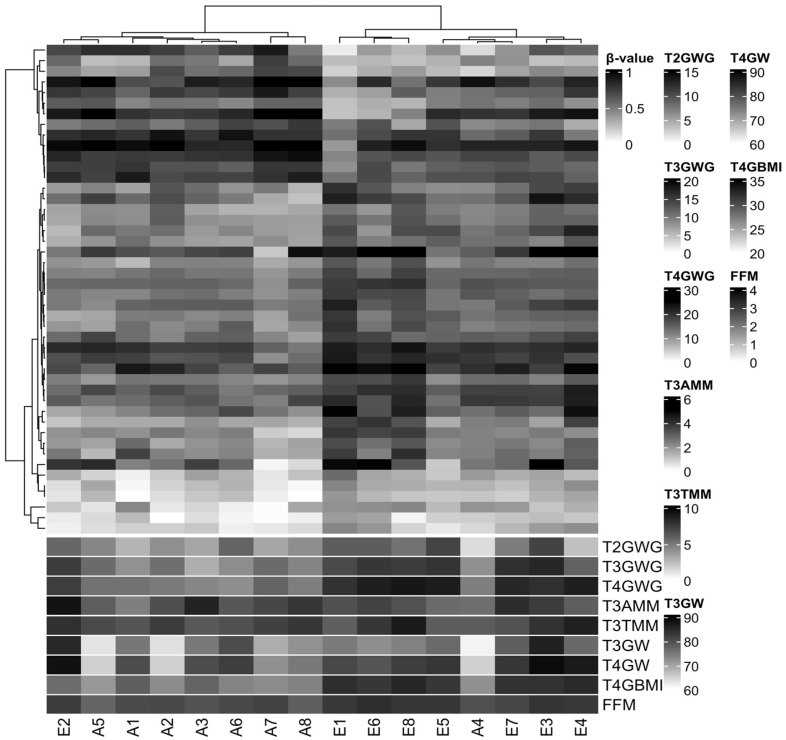
Hierarchical clustering of 16 samples based on methylation levels at 46 differentially methylated CpG sites. Pearson correlation distance with complete linkage. Heatmap colours refer to methylation levels: unmethylated (white), partially methylated (gray), and methylated (black). The heatmap columns are annotated with biological covariates correlated with principal components of the methylation data. T2GWG: gestational weight gain (kg) at T2; T3GWG: gestational weight gain (kg) at T3; T4GWG: gestational weight gain (kg) at T4; T3AMM: arm muscle mass (cm^3^) at T3; T3TMM: thigh muscle mass (cm^3^) at T3; T3GW: gestational weight (kg) at T3; T4GW: gestational weight (kg) at T4; T4GBMI: gestational body mass index (kg/m^2^) at T4; FMM: fat-free mass (%) at T5. E represents samples from the group with excessive gestational weight gain and A represents samples from the group with adequate gestational weight gain.

We did not identify differences in DMPs between the EGWG and AGWG groups at the CpGs after Benjamini–Hochberg correction (adjusted *p*-value < 0.05). This finding is not unexpected considering three factors: small sample size, population of healthy pregnant women, and mild DNAm changes. However, we found 46 CpGs (33 hyper- and 13 hypomethylated) with a *p*-value < 0.05 and DNAm differences greater than 10% between EGWG and AGWG (Table S1). Hierarchical clustering based on these DMPs identified two groups: one group of seven women who gained adequate weight and one woman who gained excessive weight, and a second group containing seven women who gained excessive weight and one woman who gained adequate weight (Figure 2). Likewise, the significant biological covariates identified through SVD analysis showed slightly different patterns for each group (Figure S3), suggesting that these patterns may be related to the epigenetic signature.

Furthermore, we identified 11 DMRs between EGWG and AGWG (DMR1 = chr6:29648161-29648756, DMR2 = chr6:31148332-31148666, DMR3 = chr7:27183133-27183816, DMR4 = chr10:530635-531584, DMR5 = chr22:51016386-51016950, DMR6 = chr16:3062296-3062975, DMR7 = chr5:110062539-110062837, DMR8 = chr17:41278135-41278906, DMR9 = chr2:27301195-27301943, DMR10 = chr5:170288671-170288788, and DMR11 = chr12:52281482-52281997), with 9 of them being hyper- and 2 being hypomethylated (Table S2) located in 13 genes (*EMILIN1*, *HOXA5*, *CPT1B*, *CLDN9*, *ZFP57*, *BRCA1*, *POU5F1*, *ANKRD33*, *HLA-B*, *RANBP17*, *ZMYND11*, *DIP2C*, *and TMEM232*) (Figure S4). The methylation status for each of the 11 DMRs revealed subtle changes in DNAm across genomic regions. Detailed information on the methylation status of these 11 DMRs, along with the corresponding heatmap, can be found in Figures S5a–k and S6. Nine human phenotypes were enriched for these DMRs (Table 3).

### 3.4. DNA Methylation Changes Are Associated with Some Foetal and Neonatal Outcomes

We explored the associations of mean maternal DNAm levels in each DMR with the extent of maternal GWG and foetal and neonatal body composition. Table 4 shows significant associations between mean maternal DNAm levels in each DMR and foetal and neonatal body composition, even after adjusting for confounding factors. Maternal DMR2 methylation was associated with foetal total thigh tissue at T2 (*p* = 0.014) and T3 (*p* = 0.018), thigh muscle mass at T2 (*p* = 0.021) and T3 (*p* = 0.032), and subcutaneous thigh fat at T2 (*p* = 0.029). There were associations of maternal DMR6 methylation with foetal total thigh tissue at T2 (*p* = 0.039), subcutaneous thigh fat at T2 (*p* = 0.017), total arm tissue at T3 (*p* = 0.002), and subcutaneous arm fat at T3 (*p* = 0.010). Maternal DMR10 methylation was associated with foetal subcutaneous thigh fat at T3 (*p* = 0.033). At T5, associations were found between maternal DMR2 methylation and neonatal fat mass percentage (*p* = 0.039) and fat mass (*p* = 0.040).

## 4. Discussion

In this study, we evaluated the influence of maternal weight gain during pregnancy on DNAm patterns and its potential impact on foetal and neonatal body composition. A rigorous selection was applied to include only healthy pregnant women in the EGWG and AGWG groups, who started gestation with a normal BMI and similar pre-pregnancy lean mass and fat mass, in order to eliminate unwanted methylation patterns related to any of the exclusion factors.

There was a difference in GWG between the two groups of women from T3 onwards. The mean difference in weight gain was approximately 6 kg. In contrast to other studies, we considered weight gain as a variable of interest and controlled for other comorbidities, like obesity [9]. There were no differences in the characteristics of the pregnant women or neonate sex between groups. Foetal body composition did not differ significantly between the EGWG and AGWG groups. Few studies have assessed adiposity via ultrasound during the foetal period, especially the effect of GWG on foetal adiposity parameters such as SCFT, which was higher in foetuses of pregnant women with alterations in the glycaemic index [21] and with obesity [22]. In our study, neonates born to EGWG women had a significantly higher weight and fat mass than those born to AGWG women. The explanation for the different fat mass results between neonates in the EGWG and AGWG groups, but not between foetuses, may be related to the gap of 6 weeks between T3 and T4, when the foetuses probably gained more weight; these time points correspond to the periods when the groups of women started to show statistically significant differences in GWG, in addition to the epigenetic marks that may be registered in the parameters of body composition of the foetus and manifested at birth.

The differences in DNAm between the EGWG and AGWG groups were mild compared to those observed in other diseases such as obesity. Studies have shown a positive association of higher methylation with a BMI outside the normal range [9]. However, we demonstrate that, even in the absence of other risk factors, EGWG can potentially trigger changes in clinical and epigenetic factors in pregnant women and their offspring. Additionally, we observed DNAm alterations in regions associated with 13 significant genes, and the methylation status of the 11 DMRs revealed subtle changes in DNAm across these genomic regions. The methylation in these genes has been less studied; we therefore highlight below the literature findings regarding the involvement of these genes in metabolism.

The levels of the elastin microfibril interfacer 1 (*EMILIN1*) gene that encodes an extracellular matrix glycoprotein were found to be altered in people with hypertension and obesity [23]. Homeobox a5 (*HOXA5*), which encodes a developmental transcription factor and is expressed in embryonic adipose tissue, is involved in adipose tissue differentiation, browning of white adipose tissue, and regulation of brown adipose tissue development [24]. The carnitine palmitoyl transferase 1b (*CPT1b*) gene controls β-oxidation by regulating the transport of long-chain fatty acids across mitochondrial membranes. Low *CPT1b* levels contribute to fat accumulation. This gene showed lower expression in the outer mitochondrial membrane muscle of obese subjects compared to lean individuals [25].

The claudin-9 (*CLDN9*) gene was differentially co-expressed in a study of obesity-associated networks in human subcutaneous adipose tissue [26]. This gene is also involved in mechanisms underlying dietary modulation of intestinal permeability with probiotics [27]. The zinc finger protein 57 homolog (*ZFP57*) gene, a transcriptional repressor, is involved in genomic imprinting and mutations in this gene have been associated with transient neonatal diabetes mellitus [28]. The *ZFP57* genes was one of 38 genes potentially associated with monogenic diabetes in a next-generation sequencing study [29]. The breast cancer 1 (*BRCA1*) gene, a tumour suppressor, has been associated with ovarian and breast cancers in women. Obesity can change the expression of this gene [30]. The POU class 5 homeobox 1 (*POU5F1*) gene, a transcription factor involved in the self-renewal of undifferentiated stem cells and induction of embryonic pluripotency via metabolic mechanisms, has been shown to be involved in β-cell dedifferentiation in type 2 diabetes [31] and is altered in breast cancer [32].

The ankyrin repeat domain 33 (*ANKRD33*) gene was found among the top 20 differently expressed genes in the placenta of women with pre-eclampsia when compared to those with normal pregnancy [33]. This gene predominated in methylation quantitative trait loci in a functional genomics study of the paediatric obese asthma phenotype [34]. The major histocompatibility complex gene class I, B (HLA-B) plays a role in the immune response to viruses and infectious diseases. Its alleles have been strongly associated with obesity because they are related to increased BMI in adults [35]. The RAN-binding protein 17 (*RANBP17*) gene, a nuclear transport receptor, has been associated with BMI and visceral adiposity in polymorphism studies [36].

The zinc finger MYND-type containing 11 (*ZMYND11*) gene plays a role in cancer and a recent transcriptome meta-analysis in young and older humans showed an inverted expression profile of this gene in resistance training [37]. The disco-interacting protein 2 homolog C (*DIP2C*) gene has been implicated in developmental delays [38]. This gene is also found among the main differentially expressed genes in polycystic ovary syndrome [39]. The transmembrane protein 232 (*TMEM232*) gene has been associated with lung diseases such as asthma [40].

In summary, the genes containing DMRs found in the present study are implicated in diabetes, hypertension, obesity, lung diseases, cancer, inflammation, adipogenesis, genomic imprinting, and lipid metabolism. Supporting our findings, several terms related to metabolism were also identified among the enriched human phenotypes, such as transient neonatal diabetes mellitus, neonatal insulin-dependent diabetes mellitus, insulin-resistant diabetes mellitus, insulin resistance, and hyperglycaemia, indicating a tenuous alteration in the metabolism of women who gained excessive weight during pregnancy and a risk pattern for developing diseases in pregnancy or later in life.

The Developmental Origins of Health and Disease (DOHaD) shows how the environment, early in life, can influence the risk of chronic diseases from childhood to adulthood [6]. DNAm, among other epigenetic modifications, are involved in mediating the relationship between the intrauterine environment and events in later life [41]. DNAm is a mechanism involving the transfer of a methyl group onto the C5 position of the cytosine to form 5-methylcytosine. It is considered a plastic and stable mechanism [42] that has been used in foetal programming studies to assess the associations of maternal and offspring metabolic health [7,9,41]. Fat mass measured via plethysmography in preschool children from the European Childhood Obesity Project was associated with their DNAm [43]. Overweight in children can be attributed to GWG [44]. A study conducted in Brazil showed that the higher the GWG, the greater the body fat mass at 6 years of age [45]. Fat mass development and expansion originate in the intrauterine period, mainly in the third trimester of pregnancy, and extend throughout the lifetime [46]. Advances in understanding the epigenomic regulation of adipogenesis reveal critical roles of DNAm in adipocyte differentiation [47]. Adipocytes go through two stages of differentiation. In the first stage, epigenetic events occur to ensure that pre-adipocytes can reach the second stage, when they acquire characteristics of mature adipocytes [48]. This process may require integration between signalling pathways involving more than 2000 transcriptional regulatory factors [49]. A previous study showed that CpG methylation increased DNA binding of the *C/EBPα* transcription factor, an important protein required for activating the differentiation of various cell types. *C/EBPα*, for example, can induce adipogenesis through *PPARγ* [50]. Induced changes in DNAm patterns may persist after elimination of the stimulus. These persistent induced changes result in a mitotically heritable cellular memory, in this case, from mother cells to daughter cells, which may contribute to diseases later in life. All these findings suggest that GWG, as an environmental factor, can alter maternal DNAm, which mediates changes in offspring phenotype, particularly in relation to fat mass.

In the present study, combined analysis revealed associations between the mean level of maternal DNAm (mainly in three DMRs) and the foetal body composition parameters investigated total thigh tissue at T2 and T3, thigh muscle mass at T2 and T3, subcutaneous thigh fat at T2 and T3, total arm tissue at T3, and subcutaneous arm fat at T3. Furthermore, there were associations between the mean level of maternal DNAm (mainly in three DMRs) and neonatal (T5) fat mass percentage and fat mass. These results suggest that body composition is not only affected by immediate circumstances but can be programmed by intrauterine exposures. This is an important finding since fat mass and fat-free mass can have different effects on health outcomes [51]. Several studies have shown that DNAm may be associated with birth weight, or parameters linked to obesity later in life [7,41,44]. To the best of our knowledge, this is the first study to demonstrate associations between DNAm with various parameters to assess body composition during both the foetal and neonatal periods.

However, we cannot overlook the existence of possible upstream effects in the results, given that maternal genetic factors were not analysed. It may be possible, for example, that maternal genetic differences in genes encoding methylases or DNA demethylase enzymes could alter observed DNAm patterns and changes in weight gain. Furthermore, it has been described that paternal obesity may have an effect on foetal development, showing the influence of the father on foetal programming [52]. Therefore, experiments involving genetic factors could be conducted to determine the influence of these factors on GWG, and despite not being part of the objective of this study, the inclusion of paternal variables as confounding factors would be interesting to assess the phenotype changes in the offspring.

We point out some limitations of this study including the (1) DNAm changes which were assessed at the end of pregnancy and not compared to DNAm patterns at baseline, although the study design has the advantage that the whole population starts pregnancy with an adequate BMI; (2) the small sample size; (3) investigation of the offspring DNAm; and (4) the lack of assessment of gene expression that could be correlated with the methylation levels in DMRs. Thus, further cohort studies are necessary to confirm our results in different human populations and to elucidate the mechanistic links of our current findings.

## 5. Conclusions

To our knowledge, this is the first study that assessed the relationship of maternal DNAm in EGWG with foetal and neonatal adiposity. The methylation pattern in the EGWG women who started pregnancy with a normal BMI indicated a risk for developing chronic diseases and involvement of maternal DNAm in foetal lean and fat mass and in neonatal fat mass. These findings provide support for possible epigenetic programming of offspring body composition and contribute to the literature data that link specific exposures to variations in epigenetic profiles and metabolic phenotypes in humans.

## Figures and Tables

**Table 1:** Characteristics of the pregnant women with adequate/excessive gestational weight gain and their neonates.

Variables	Excessive Gestational Weight Gain (*n* = 30)	Adequate Gestational Weight Gain (*n* = 45)	*p*
Pregnant women			
Age (years)	25.9 ± 6.0	29.0 ± 6.4	0.064
Ethnicity			
White	10 (33.3%)	15 (33.3%)	0.351
Black	03 (10.0%)	10 (22.2%)	
Brown	17 (56.7%)	20 (44.4%)	
Marital status			
Single/without partner	00 (0.0%)	04 (8.8%)	0.245
Married/with partner	30 (100.0%)	41 (91.1%)	
Education			
Elementary school	01 (3.3%)	09 (20.0%)	0.115
High school degree	24 (80.0%)	30 (66.7%)	
University degree	05 (16.7%)	06 (13.3%)	
Parity			
0	05 (16.7%)	07 (15.6%)	0.991
1	19 (63.3%)	29 (64.4%)	
2 a 4	06 (20.0%)	09 (20.0%)	
Neonates			
Age (weeks)	39.9 ± 1.1	39.2 ± 1.4	0.162
Sex			
Female	17 (56.6%)	16 (35.6%)	0.071
Male	13 (43,3%)	29 (64.4%)	

Mean ± SD or number of individuals (percentage). *T* test for independent samples or chi-square test.

**Table 2:** Anthropometry and body composition of the pregnant women and of their foetuses and neonates.

Variables	Excessive Gestational Weight Gain (*n* = 30)		Adequate Gestational Weight Gain (*n* = 45)		*p*
Pregnant women	Mean	SD	Mean	SD	
T1 Pre-pregnancy weight (kg)	60.54	6.37	57.52	7.17	0.682
T1 Height (cm)	163.71	6.56	161.39	6.93	0.785
T1 Pre-pregnancy BMI (kg/m^2^)	22.68	1.74	22.06	1.79	0.405
T4 BMI (kg/m^2^)	29.90	2.01	27.15	1.84	0.010
T4 Total gestational weight gain (kg)	19.60	2.43	13.26	1.54	0.010
Pre-pregnancy fat mass (%)	29.32	4.03	26.80	4.85	0.262
Pre-pregnancy fat mass (kg)	18.75	4.19	15.82	4.40	0.167
Pre-pregnancy fat-free body mass (kg)	44.17	4.60	42.26	3.85	0.188
Pre-pregnancy muscle mass (kg)	41.94	4.35	40.04	3.58	0.135
Foetuses	Mean	SD	Mean	SD	
T2 Foetal weight (g)	629.30	204.02	598.87	186.40	0.875
T3 Foetal weight (g)	2172.82	353.43	2132.18	457.47	0.514
T2 SCFT (mm)	2.84	0.52	2.95	0.56	0.721
T3 SCFT (mm)	4.13	0.76	4.07	1.07	0.521
T2 Total thigh tissue (cm^3^)	5.23	1,78	5.12	1.53	0.945
T3 Total thigh tissue (cm^3^)	13.37	2.97	13.53	3.25	0.955
T2 Thigh muscle mass (cm^3^)	2.97	1.04	2.90	0.93	0.729
T3 Thigh muscle mass (cm^3^)	7.69	1.72	7.54	1.80	0.643
T2 Subcutaneous thigh fat (cm^3^)	2.26	0.84	2.27	0.76	0.991
T3 Subcutaneous thigh fat (cm^3^)	5.68	1.64	6.03	1.61	0.225
T2 Total arm tissue (cm^3^)	3.05	0.93	2.85	0.83	0.358
T3 Total arm tissue (cm^3^)	7.01	1.55	7.07	1.87	0.860
T2 Arm muscle mass (cm^3^)	1.57	0.50	1.46	0.46	0.224
T3 Arm muscle mass (cm^3^)	3.45	0.82	3.52	0.98	0.683
T2 Subcutaneous arm fat (cm^3^)	1.46	0.52	1.46	0.63	0.991
T3 Subcutaneous arm fat (cm^3^)	3.55	0.93	3.56	1.02	0.928
Neonates	Mean	SD	Mean	SD	
T5 Weight (g)	3354.87	298.47	3068.50	386.57	0.027
T5 Length (cm)	50.03	1.78	48.80	2.33	0.182
T5 Fat-free mass percentage (%)	90.39	3.98	91.57	5.65	0.120
T5 Fat mass percentage (%)	9.61	3.98	8.43	5.65	0.120
T5 Fat-free mass (kg)	3.08	0.19	2.76	0.27	0.218
T5 Fat mass (kg)	0.34	0.13	0.26	0.21	0.039

Mean ± SD. *T* test for independent samples. BMI: body mass index. SCFT: subcutaneous abdominal fat thickness. T1 ≤ 15 gestational weeks. T2 = 20–26 weeks. T3 = 30–36 weeks. T4 = delivery and T5 = 72 h after delivery.

**Table 3:** Enriched human phenotype ontology based on GREAT enrichment analysis of differentially methylated regions in pregnant women with adequate and excessive gestational weight gain.

Terms Name	Binom Raw *p*-Value	Binom Fold Enrichment
Transient neonatal diabetes mellitus	0.0010	1041.1
Neonatal insulin-dependent diabetes mellitus	0.0015	656.2
Severe failure to thrive	0.0037	269.7
Insulin-resistant diabetes mellitus	0.0185	53.7
Insulin resistance	0.0236	42.0
Breast carcinoma	0.0255	38.7
Neoplasm of the breast	0.0272	36.4
Hyperglycemia	0.0275	35.9
Dehydration	0.0379	25.9

**Table 4:** Multiple linear regression models to assess the associations between maternal DNAm with foetal and neonatal body composition parameters.

T2 Total thigh tissue	Β	r²	*p*	95% CI
DMR 2	9.172	0.853	0.014	2.340; 16.005
Gestational weight gain	−0.010		0.843	−0.127; 0.106
Pre-pregnancy BMI	−0.780		0.005	−1.249; −0.310
Maternal age	−0.048		0.400	−0.172; 0.076
Sex	1.026		0.092	−0.205; 2.257
Gestational age	0.833		0.002	0.411; 1.255
DMR 6	21.516	0.820	0.039	1.407; 41.625
Gestational weight gain	−0.072		0.322	−0.228; 0.084
Pre-pregnancy BMI	−0.786		0.008	−1.316; −0.256
Maternal age	−0.044		0.489	−0.181; 0.093
Sex	1.313		0.070	−0.131; 2.757
Gestational age	0.939		0.002	0.443; 1.434
T3 Total thigh tissue	Β	r²	*p*	95% CI
DMR 2	8.265	0.715	0.018	1.790; 14.740
Gestational weight gain	0.045		0.371	−0.064; 0.154
Pre-pregnancy BMI	−0.393		0.080	−0.844; 0.058
Maternal age	0.036		0.487	−0.077; 0.150
Sex	0.679		0.127	−0.235; 1.593
Gestational age	0.472		0.155	−0215; 1.160
T2 Thigh muscle mass	Β	r²	*p*	95% CI
DMR 2	5.314	0.814	0.021	1.006; 9.622
Gestational weight gain	−0.026		0.440	−0.100; 0.047
Pre-pregnancy BMI	−0.416		0.011	−0.712; −0.120
Maternal age	−0.012		0.739	−0.090; 0.066
Sex	0.773		0.051	−0.003; 1.549
Gestational age	0.431		0.005	0.165; 0.697
T3 Thigh muscle mass	Β	r²	*p*	95% CI
DMR 2	6.373	0.687	0.032	0.694; 12.052
Gestational weight gain	0.067		0.147	−0.029; 0.162
Pre-pregnancy BMI	−0.177		0.339	−0.572; 0.219
Maternal age	0.036		0.429	−0.063; 0.136
Sex	0.442		0.244	−0.360; 1.243
Gestational age	0.358		0.213	−0.246; 0.961
T2 Subcutaneous thigh fat	Β	r²	*p*	95% CI
DMR 2	3.858	0.846	0.029	0.506; 7.211
Gestational weight gain	0.016		0.549	−0.041; 0.073
Pre-pregnancy BMI	−0.364		0.006	−0.594; −0.133
Maternal age	−0.037		0.207	−0.097; 0.024
Sex	0.254		0.367	−0.350; 0.858
Gestational age	0.402		0.002	0.195; 0.609
DMR 6	10.933	0.862	0.017	2.494; 19.372
Gestational weight gain	−0.019		0.532	−0.084; 0.047
Pre-pregnancy BMI	−0.385		0.004	−0.607; −1.162
Maternal age	−0.035		0.202	−0.093; 0.023
Sex	0.424		0.148	−0.182; 1.030
Gestational age	0.463		0.001	0.255; 0.671
T3 Subcutaneous thigh fat	Β	r²	*p*	95% CI
DMR 10	7.604	0.596	0.033	0.763; 14.445
Gestational weight gain	−0.034		0.267	−0.100; 0.031
Pre-pregnancy BMI	−0.247		0.064	−0.511; 0.018
Maternal age	0.015		0.619	−0.052; 0.083
Sex	0.257		0.311	−0.284; 0.797
Gestational age	−0.004		0.982	−0.401; 0.393
T3 Total arm tissue	Β	r²	*p*	95% CI
DMR 6	−25.640	0.804	0.002	−39.368; −11.911
Gestational weight gain	0.115		0.039	0.007; 0.222
Pre-pregnancy BMI	0.410		0.043	0.016; 0.805
Maternal age	0.038		0.400	−0.059; 0.134
Sex	−0.269		0.460	−1.059; 0.521
Gestational age	0.311		0.257	−0.271; 0.893
T3 Subcutaneous arm fat	Β	r²	*p*	95% CI
DMR 6	−17.433	0.667	0.010	−29.597; −5.270
Gestational weight gain	0.078		0.097	−0.017; 0.172
Pre-pregnancy BMI	0.233		0.165	−0.116; 0.583
Maternal age	0.014		0.716	−0.071; 0.099
Sex	−0.339		0.302	−1.039; 0.361
Gestational age	0.175		0.461	−0.340; 0.691
T5 Fat mass percentage	Β	r²	*p*	95% CI
DMR 2	−20.299	0.761	0.039	−39.362; −1.236
Gestational weight gain	0.500		0.013	0.135; 0.865
Pre-pregnancy BMI	0.651		0.275	−0.615; 1.916
Maternal age	0.071		0.530	−0.175; 0.318
Sex	−5.207		0.002	−7.968; −2.446
Gestational age	−0.653		0.196	−1.710; 0.403
T5 Fat mass	Β	r²	*p*	95% CI
DMR 2	−0.719	0.780	0.040	−0.395; −0.042
Gestational weight gain	0.019		0.009	0.006; 0.032
Pre-pregnancy BMI	0.026		0.216	−0.018; 0.071
Maternal age	0.003		0.391	−0.005; 0.012
Sex	−0.180		0.002	−0.279; 0.082
Gestational age	−0.016		0.374	−0.053; 0.022

Multiple linear regression models. BMI: body mass index. T1 ≤ 15 gestational weeks. T2 = 20–26 weeks. T3 = 30–36 weeks. T4 = delivery and T5 = 72 h after delivery.

## Data Availability

The datasets supporting the conclusions of this article are included within the article and its Supplementary Materials. The raw data are not publicly available due individual privacy but are available from the corresponding author on reasonable request.

## Supplementary Materials

The following information supporting the DNA methylation analysis, including the pre-processing steps can be downloaded at https://www.mdpi.com/article/10.3390/epigenomes7030018/s1, Supplementary Figure S1: Density plot of beta-values. Left panel: Raw data. Right panel: BMIQ-normalized data; Figure S2: Multidimensional scaling of the 1% most variable CpG sites; Table S1: 46 differentially methylated CpG sites between EGWG and AGWG; Figure S3: Singular value decomposition (SVD); Table S2: Identification of differentially methylated regions; Figure S4: Chromosome location of differentially methylated regions associated with the transcription start sites of genes; Figures S5a–k: Differential methylation regions identified by bumphunter/CHAMP and Figure S6: Heatmap of average differential methylation regions beta values.

## Abbreviations

IOM: The Institute of Medicine; BMI: body mass index; GA: gestational age; GWG: gestational weight gain; AGWG: adequate gestational weight gain; EGWG: excessive gestational weight gain; SCFT: subcutaneous abdominal fat thickness; T1: time 1; T2: time 2; T3: time 3; T4: time 4; T5: time 5; DNAm: DNA methylation; CpGs: CpG sites; DMPs: differential methylated positions; DMRs: differentially methylated regions; SVD: singular value decomposition.
