# Supplementary material for: Excessive Gestational Weight Gain Alters DNA Methylation and Influences Foetal and Neonatal Body Composition
Source: Epigenomes. 2023 Aug 16;7(3):18. doi: 10.3390/epigenomes7030018 (PMC10443290; doi:10.3390/epigenomes7030018)

Figure S4: Chromosome location of differentially methylated regions associated with the transcription start sites of genes

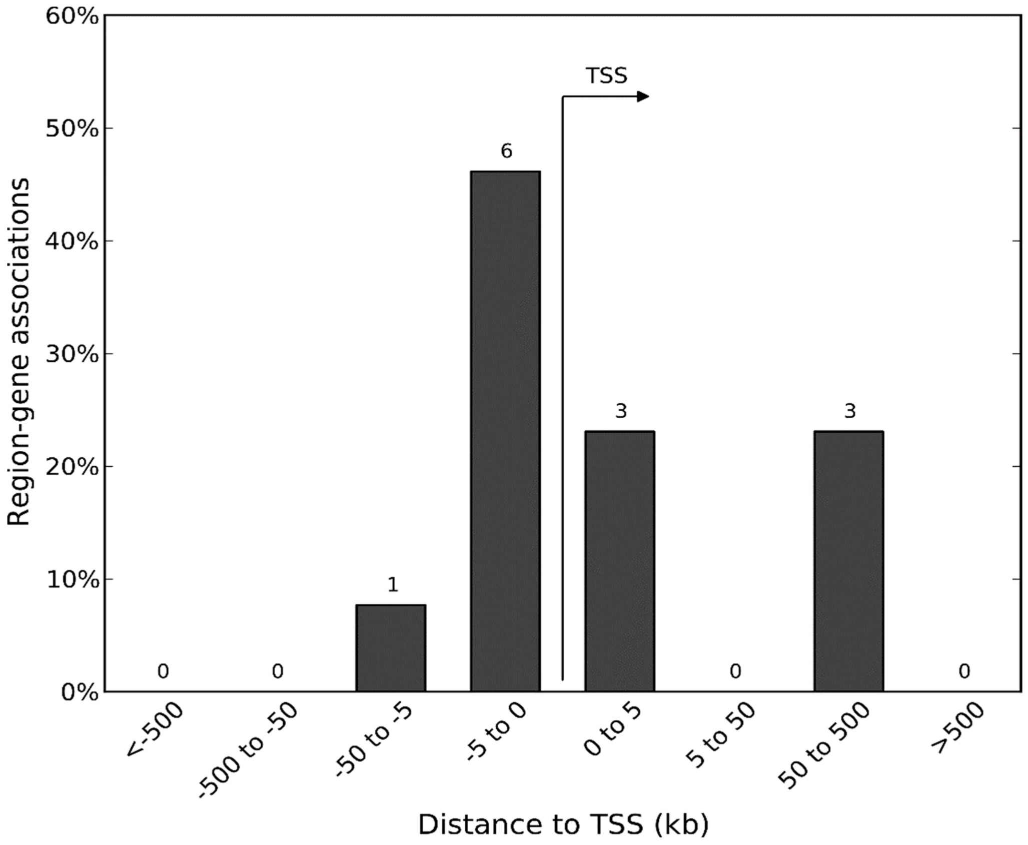

Supplement: Supplementary file 1 [file epigenomes-07-00018-s001.zip › Figure S4.pdf]
