# Supplementary material for: Excessive Gestational Weight Gain Alters DNA Methylation and Influences Foetal and Neonatal Body Composition
Source: Epigenomes. 2023 Aug 16;7(3):18. doi: 10.3390/epigenomes7030018 (PMC10443290; doi:10.3390/epigenomes7030018)
Supplement: Supplementary file 1 [file epigenomes-07-00018-s001.zip › Figures S5a¿Ck and S6.pdf]

Figures S5a-k: Differential methylation regions identified by bumphunter/CHAMP.

Figure S5(a)

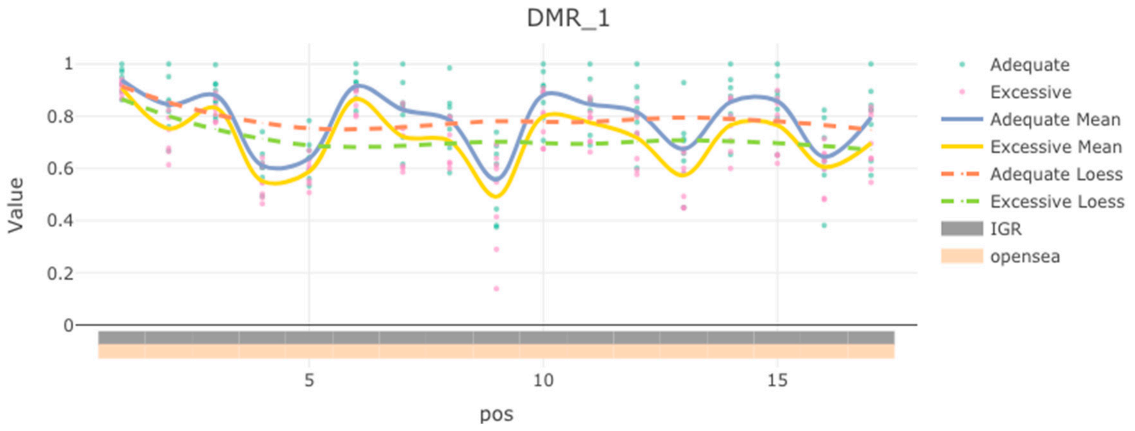

Figure S5(b)

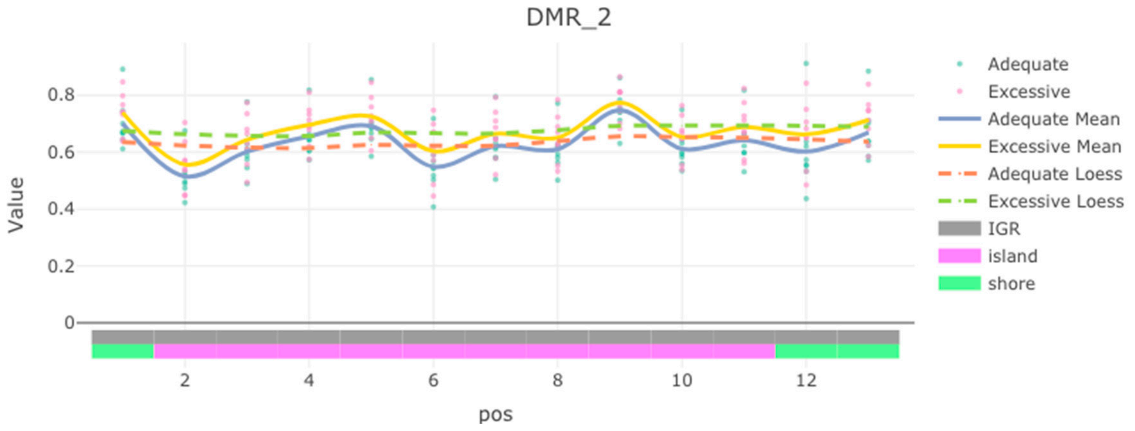

Figure S5(c)

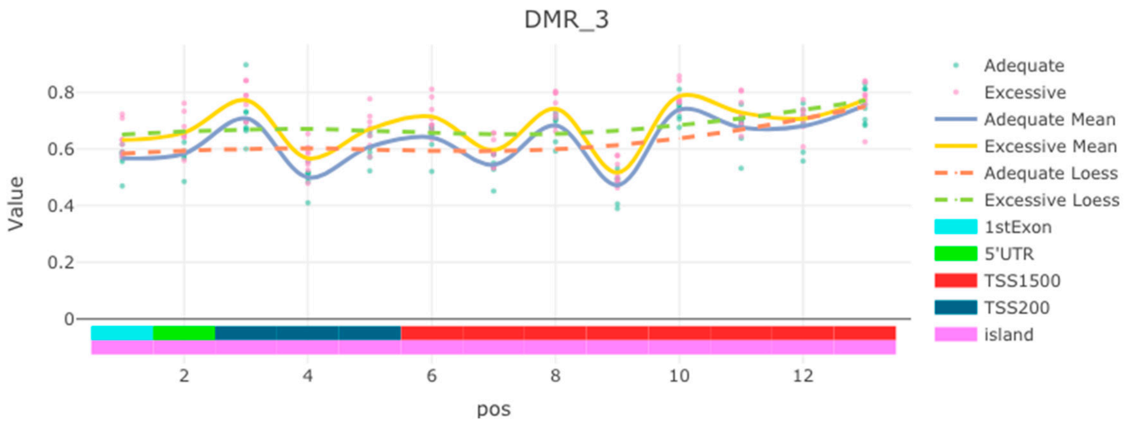

Figure S5(d)

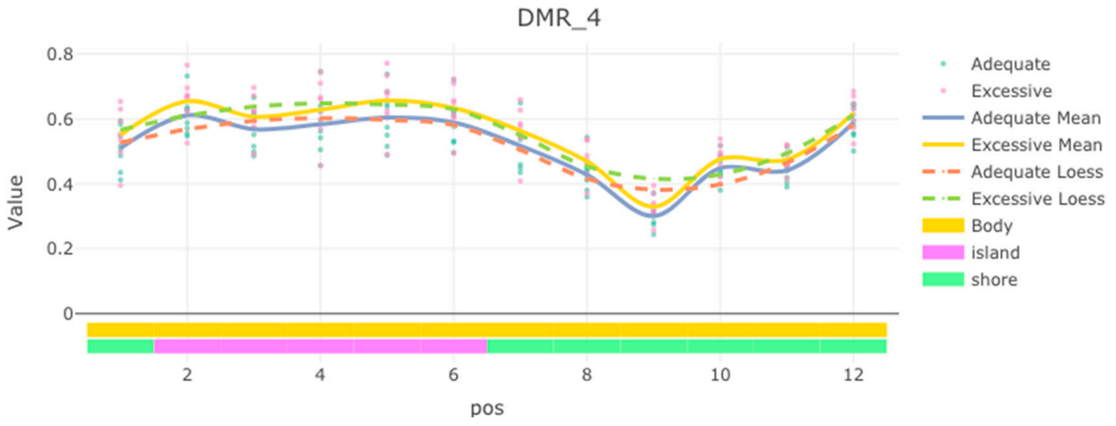

Figure S5(e)

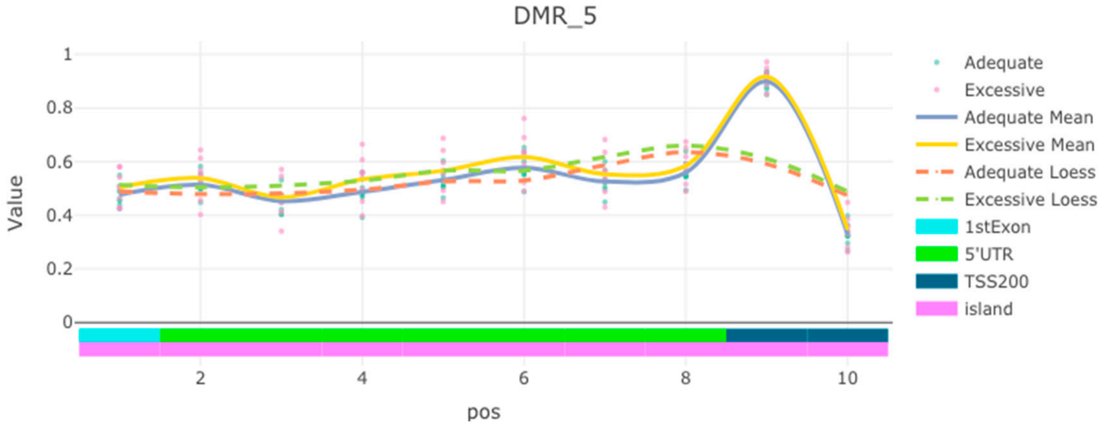

Figure S5(f)

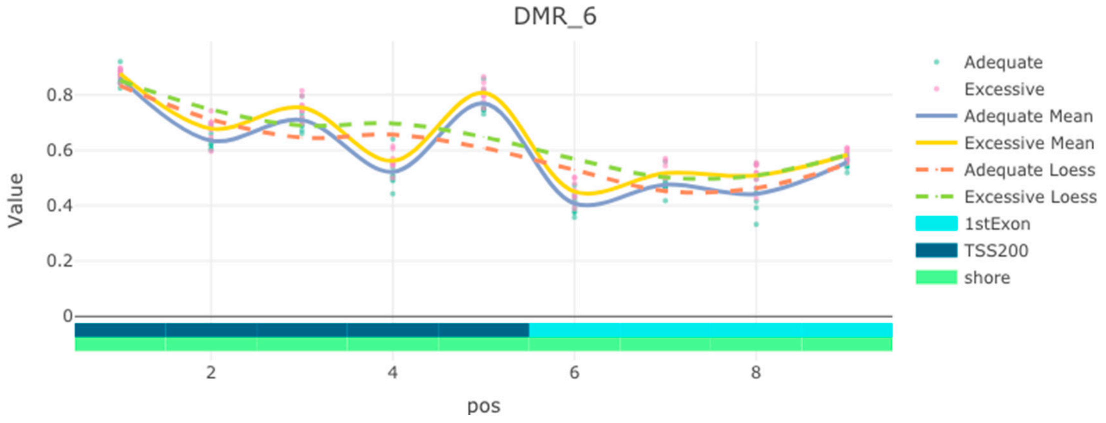

Figure S5(g)

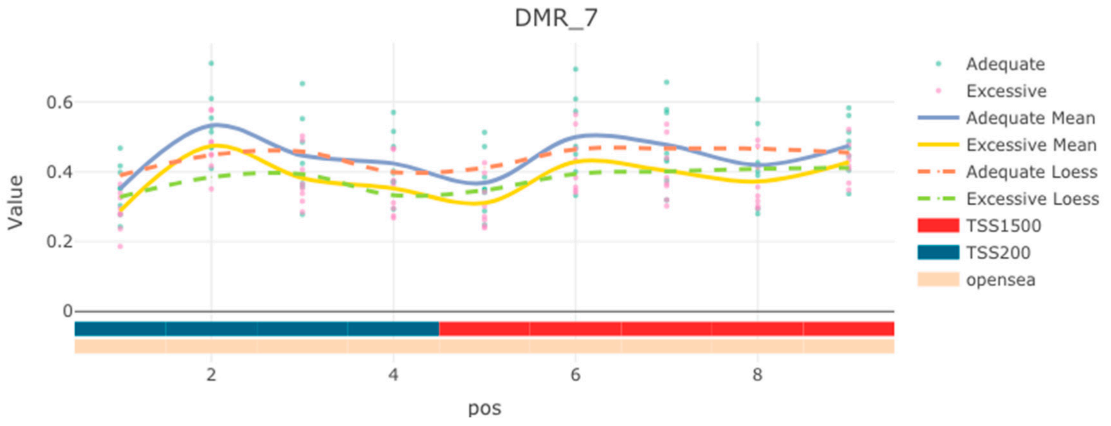

Figure S5(h)

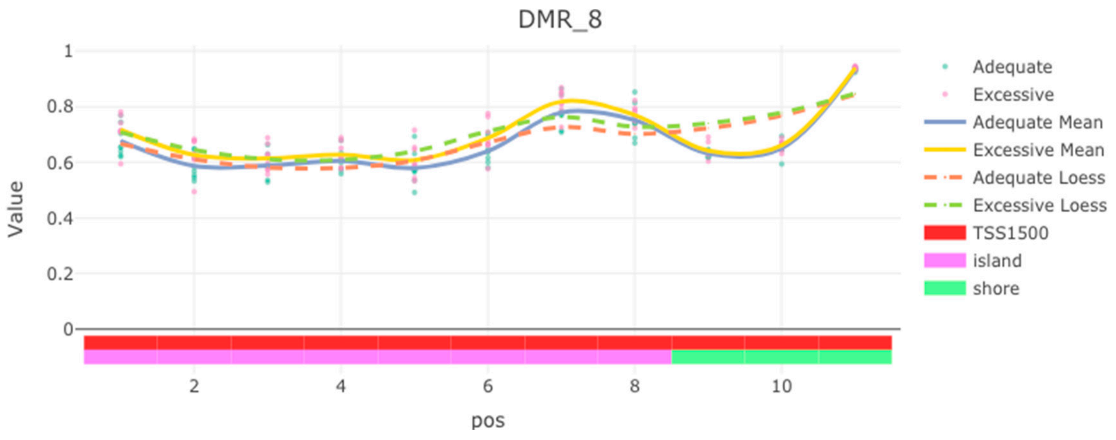

Figure S5(i)

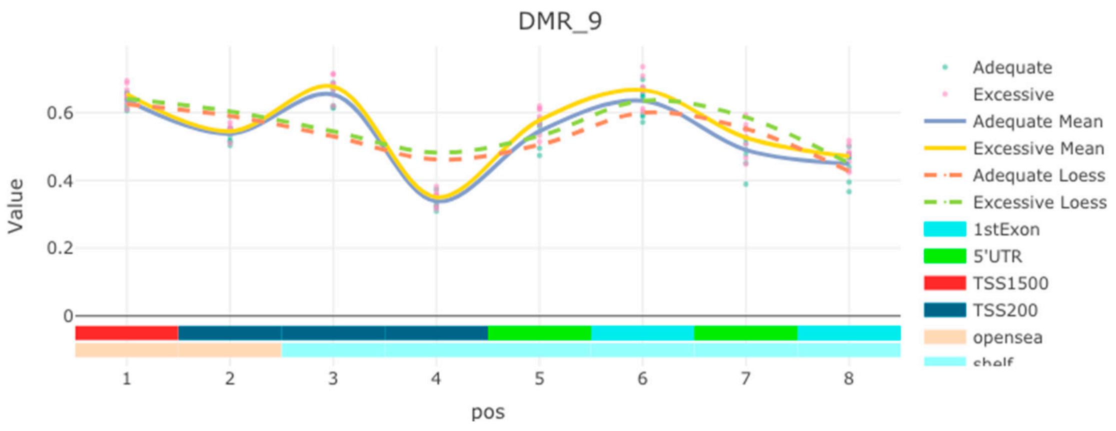

Figure S5(j)

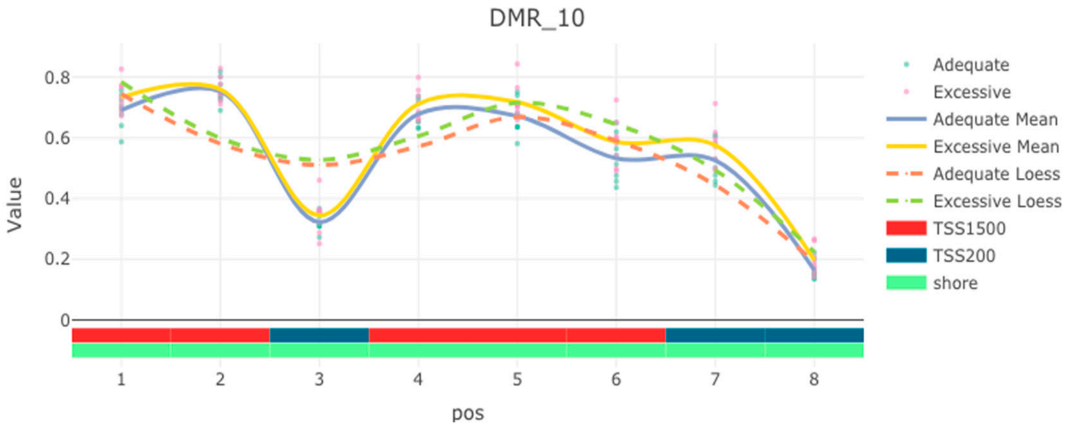

Figure S5(k)

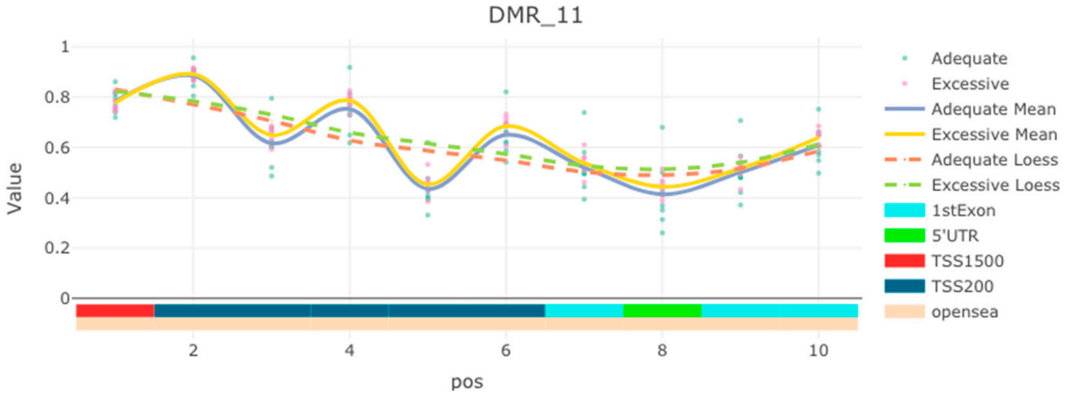

Green and pink points represent the methylation status for women from the AGWG (Adequate Gestational Weight Gain) and EGWG (Excessive Gestational Weight Gain) groups, respectively, showing the significant CpG sites identified by bumphunter. The blue and yellow lines represent the mean methylation levels of AGWG and EGWG groups, respectively. The red and green dashed lines represent the loess smoothing curves fitted to the methylation levels of AGWG and EGWG groups, respectively, helping to better visualize general methylation trends. Additionally, the CpG sites are categorized based on their location, in relation to the CpG island: island, shore, shelf, and open sea. Their associations with genetic characteristics are indicated as IGR, 1° Exon, 5'UTR, TSS1500, TSS200, body, 3'UTR.

Figure S6 - Heatmap of average differential methylation regions beta values

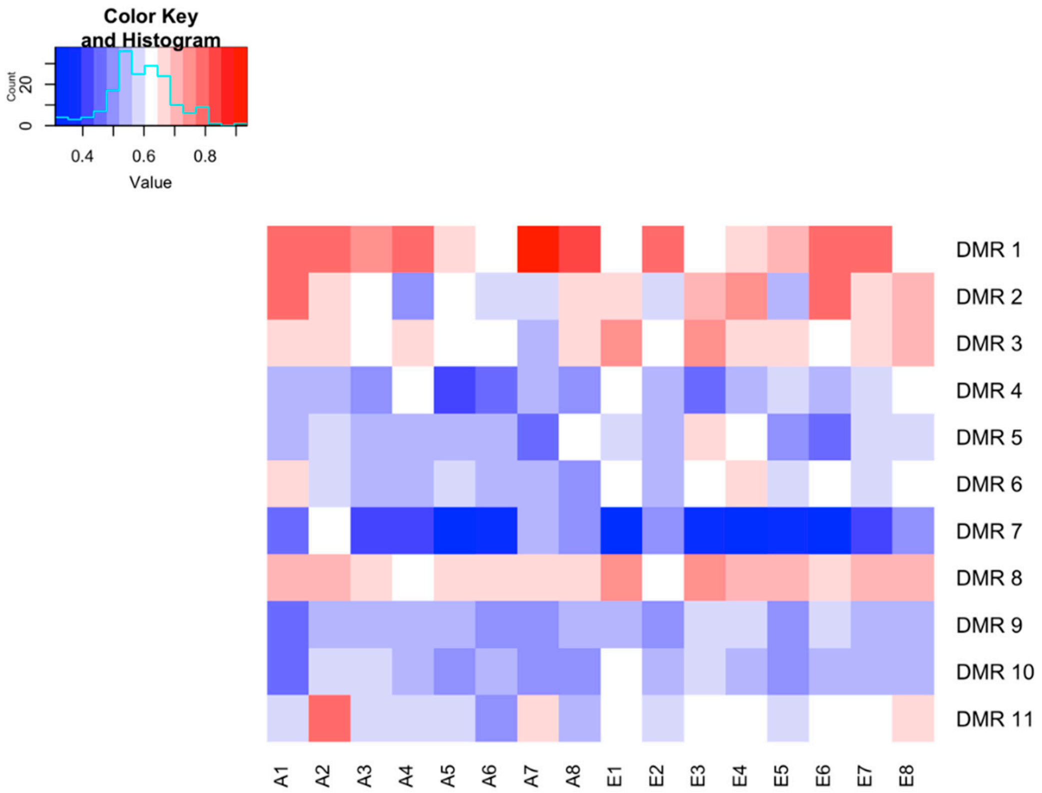

Heatmap of average DMRs beta values. DMR: Differentially methylated region. Heatmap colours refer to methylation levels: unmethylated (white), partially methylated (blue), and methylated (red). E: represent samples from the group with EGWG and A: represent samples from the group with AGWG.
