# Supplementary material for: Excessive Gestational Weight Gain Alters DNA Methylation and Influences Foetal and Neonatal Body Composition
Source: Epigenomes. 2023 Aug 16;7(3):18. doi: 10.3390/epigenomes7030018 (PMC10443290; doi:10.3390/epigenomes7030018)
Supplement: Supplementary file 1 [file epigenomes-07-00018-s001.zip › Table S1.pdf]

Tabela S1: CpG sites hyper- and hypo-methylated

| CpG ID | Average log2 methylation | t-statistics | B | p-value | Methylation difference |
|---|---|---|---|---|---|
| cg24287362 | 0.3028 | 2.9711 | -3.4578 | 0.0085 | 0.1005 |
| cg12777182 | -0.2651 | 2.9304 | -3.4855 | 0.0093 | 0.1345 |
| cg12308308 | 5.2214 | 2.8816 | -3.5186 | 0.0103 | 0.1785 |
| cg17774776 | 0.3051 | 2.8428 | -3.5451 | 0.0112 | 0.1129 |
| cg03111560 | 0.8286 | 2.7924 | -3.5796 | 0.0124 | 0.2045 |
| cg24000535 | 1.3982 | 2.7062 | -3.6385 | 0.0149 | 0.1240 |
| cg19544767 | 1.6306 | -2.7004 | -3.6425 | 0.0151 | -0.1428 |
| cg11821245 | 1.9143 | 2.6197 | -3.6976 | 0.0179 | 0.1077 |
| cg18980028 | 1.0836 | 2.6181 | -3.6987 | 0.0179 | 0.1346 |
| cg02988816 | 0.7207 | 2.5989 | -3.7118 | 0.0187 | 0.1011 |
| cg21177396 | -0.1893 | 2.5551 | -3.7417 | 0.0204 | 0.1709 |
| cg03004329 | 0.1586 | 2.5226 | -3.7639 | 0.0218 | 0.1139 |
| cg05907464 | 0.9066 | -2.5096 | -3.7727 | 0.0224 | -0.1041 |
| cg21598751 | 4.8112 | -2.4887 | -3.7869 | 0.0234 | -0.1479 |
| cg18896979 | 0.6588 | 2.4848 | -3.7895 | 0.0236 | 0.1219 |
| cg02715879 | 1.1229 | 2.4829 | -3.7908 | 0.0237 | 0.1085 |
| cg13290274 | -0.0986 | 2.4257 | -3.8297 | 0.0266 | 0.1056 |
| cg13294447 | -0.3781 | 2.3961 | -3.8497 | 0.0283 | 0.1829 |
| cg23686831 | -0.2760 | 2.3891 | -3.8545 | 0.0287 | 0.1018 |
| cg00180531 | 5.3431 | -2.3847 | -3.8574 | 0.0289 | -0.1064 |
| cg01827501 | -1.8938 | 2.3697 | -3.8675 | 0.0298 | 0.1204 |
| cg19384241 | 4.5697 | -2.362 | -3.8727 | 0.0303 | -0.2001 |
| cg14503564 | 0.0628 | 2.3456 | -3.8838 | 0.0313 | 0.1196 |
| cg06588529 | 1.7292 | -2.3304 | -3.894 | 0.0323 | -0.1199 |
| cg17513925 | -1.7726 | 2.3237 | -3.8985 | 0.0327 | 0.1227 |
| cg14157578 | -0.1890 | -2.2888 | -3.9219 | 0.0351 | -0.1133 |
| cg24399712 | -2.8670 | 2.2775 | -3.9295 | 0.0359 | 0.1016 |
| cg22035305 | 1.4000 | -2.2707 | -3.9341 | 0.0364 | -0.1012 |
| cg11407226 | 0.0803 | 2.2582 | -3.9424 | 0.0373 | 0.1158 |
| cg00154902 | 0.0230 | 2.2552 | -3.9444 | 0.0375 | 0.1462 |
| cg03635442 | -2.4433 | 2.2398 | -3.9547 | 0.0387 | 0.1009 |
| cg00570954 | 0.5327 | 2.2342 | -3.9584 | 0.0391 | 0.1035 |
| cg07837187 | -0.1968 | 2.2183 | -3.9690 | 0.0403 | 0.1007 |
| cg01713812 | 4.0218 | 2.2137 | -3.9721 | 0.0407 | 0.1471 |
| cg13046221 | -0.3610 | 2.2062 | -3.9770 | 0.0413 | 0.1033 |
| cg06087988 | 0.9663 | 2.2060 | -3.9771 | 0.0413 | 0.1817 |
| cg19774973 | -1.5532 | 2.2054 | -3.9775 | 0.0414 | 0.1003 |
| cg05765605 | -0.7449 | -2.2050 | -3.9778 | 0.0414 | -0.1438 |
| cg24851651 | 2.4423 | 2.1687 | -4.0018 | 0.0445 | 0.2470 |
| cg18263335 | 0.8711 | -2.1640 | -4.0049 | 0.0449 | -0.1502 |
| cg14317533 | -0.5041 | -2.1460 | -4.0168 | 0.0465 | -0.1668 |
| cg20124410 | 0.2895 | -2.1452 | -4.0174 | 0.0466 | -0.1179 |
| cg21109666 | 0.0986 | 2.1442 | -4.0180 | 0.0467 | 0.1331 |
| cg22443212 | 0.2360 | -2.1253 | -4.0305 | 0.0484 | -0.2231 |
| cg20975419 | -2.1580 | 2.1186 | -4.0348 | 0.0491 | 0.1334 |
| cg19585676 | 0.2001 | 2.1089 | -4.0412 | 0.0500 | 0.1453 |
