# Supplementary material for: Excessive Gestational Weight Gain Alters DNA Methylation and Influences Foetal and Neonatal Body Composition
Source: Epigenomes. 2023 Aug 16;7(3):18. doi: 10.3390/epigenomes7030018 (PMC10443290; doi:10.3390/epigenomes7030018)
Supplement: Supplementary file 1 [file epigenomes-07-00018-s001.zip › Table S2.pdf]

Table S2: Identification of differentially methylated regions (DMRs)

DMRs	Gene names	Chromosome	DMR start	DMR end	DMR value
DMR 1	*ZFP57*	chr6	29648161	29648756	-1.541390348
DMR2	*POU5F1*	chr6	31148332	31148666	3.091541791
DMR2	*HLA-B*	chr6	31148332	31148666	3.091541791
DMR 3	*HOXA5*	chr7	27183133	27183816	2.880343196
DMR4	*ZMYND11*	chr10	530635	531584	2.33109591
DMR4	*DIP2C*	chr10	530635	531584	2.33109591
DMR 5	*CPT1B*	chr22	51016386	51016950	1.87784786
DMR 6	*CLDN9*	chr16	3062296	3062975	1.841206709
DMR 7	*TMEM232*	chr5	110062539	110062837	-1.839050648
DMR 8	*BRCA1*	chr17	41278135	41278906	1.649115948
DMR 9	*EMILIN1*	chr2	27301195	27301943	1.838495437
DMR 10	*RANBP17*	chr5	170288671	170288788	1.739944579
DMR 11	*ANKRD33*	chr12	52281482	52281997	1.32087910
