# Supplementary figures and images for: Excessive Gestational Weight Gain Alters DNA Methylation and Influences Foetal and Neonatal Body Composition
Source: Epigenomes. 2023 Aug 16;7(3):18. doi: 10.3390/epigenomes7030018 (PMC10443290; doi:10.3390/epigenomes7030018)

Figure S1. Density plot of beta-values. Left panel: Raw data. Right panel: BMIQ-normalized data

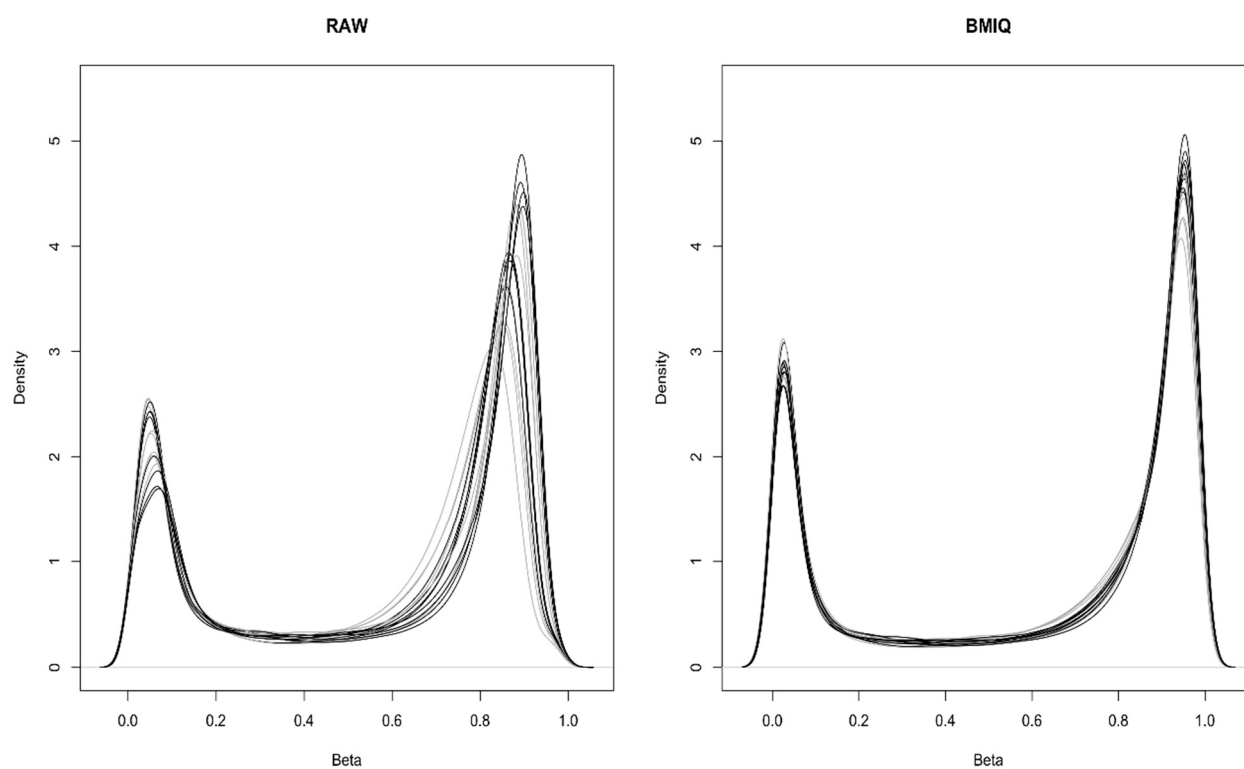

Supplement: Supplementary file 1 [file epigenomes-07-00018-s001.zip › Figure S1.pdf]

Figure S2: Multidimensional scaling of the 1% most variable CpG sites

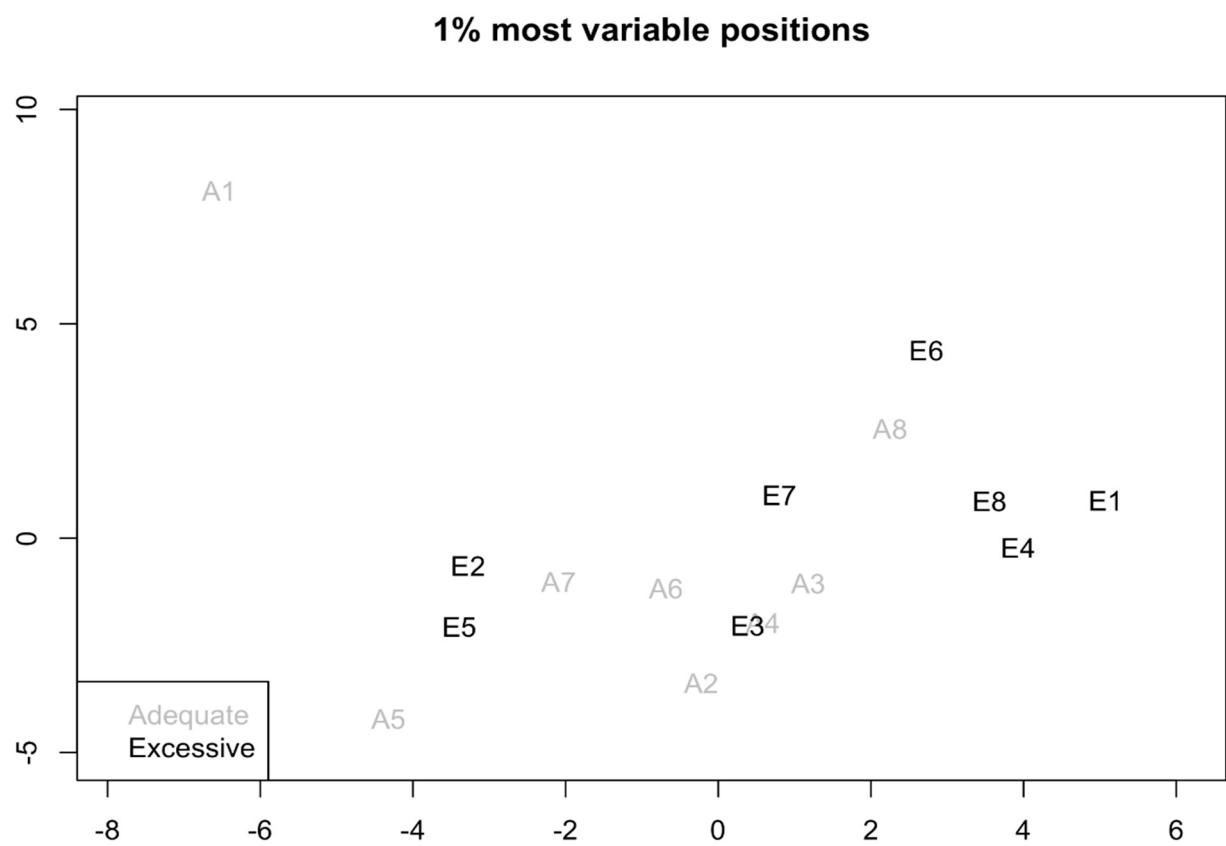

Supplement: Supplementary file 1 [file epigenomes-07-00018-s001.zip › Figure S2.pdf]

Figure S3: Singular value decomposition (SVD)

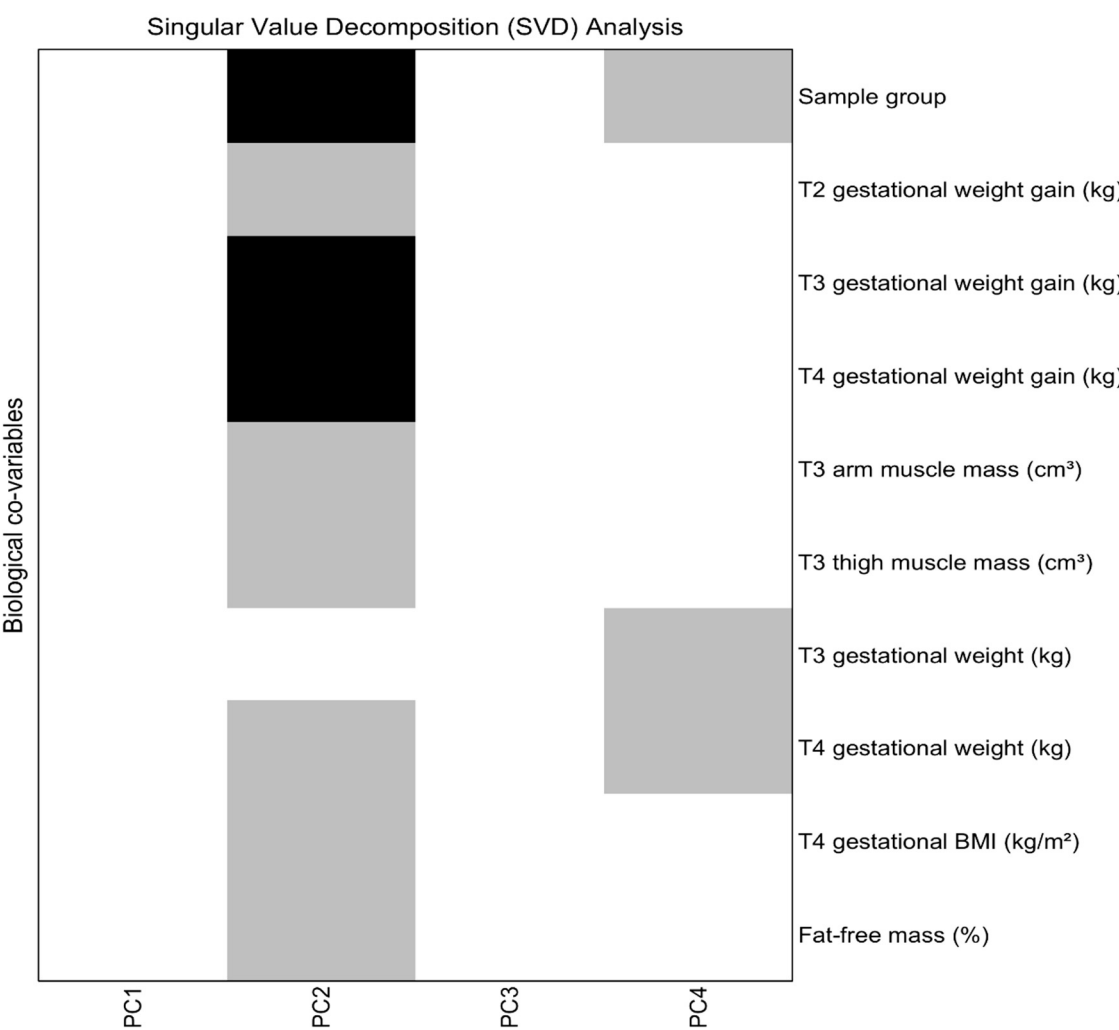

Supplement: Supplementary file 1 [file epigenomes-07-00018-s001.zip › Figure S3.pdf]
